# SLC26A6-selective inhibitor identified in a small-molecule screen blocks fluid absorption in small intestine

**DOI:** 10.1172/jci.insight.147699

**Published:** 2021-06-08

**Authors:** Onur Cil, Peter M. Haggie, Joseph-Anthony Tapia Tan, Amber A. Rivera, Alan S. Verkman

**Affiliations:** 1 Department of Pediatrics and; 2 Departments of Medicine and Physiology, University of California, San Francisco, San Francisco, California, USA.

**Keywords:** Gastroenterology, Therapeutics, Epithelial transport of ions and water

## Abstract

SLC26A6 (also known as putative anion transporter 1 [PAT1]) is a Cl^–^/HCO_3_^–^ exchanger expressed at the luminal membrane of enterocytes where it facilitates intestinal Cl^–^ and fluid absorption. Here, high-throughput screening of 50,000 synthetic small molecules in cells expressing PAT1 and a halide-sensing fluorescent protein identified several classes of inhibitors. The most potent compound, the pyrazolo-pyrido-pyrimidinone PAT1_inh_-B01, fully inhibited PAT1-mediated anion exchange (IC_50_ ~350 nM), without inhibition of the related intestinal transporter SLC26A3 (also known as DRA). In closed midjejunal loops in mice, PAT1_inh_-B01 inhibited fluid absorption by 50%, which increased to >90% when coadministered with DRA inhibitor DRA_inh_-A270. In ileal loops, PAT1_inh_-B01 blocked fluid absorption by >80%, whereas DRA_inh_-A270 was without effect. In colonic loops, PAT1_inh_-B01 was without effect, whereas DRA_inh_-A270 completely blocked fluid absorption. In a loperamide constipation model, coadministration of PAT1_inh_-B01 with DRA_inh_-A270 increased stool output compared with DRA_inh_-A270 alone. These results provide functional evidence for complementary and region-specific roles of PAT1 and DRA in intestinal fluid absorption, with PAT1 as the predominant anion exchanger in mouse ileum. We believe that PAT1_inh_-B01 is a novel tool to study intestinal ion and fluid transport and perhaps a drug candidate for small intestinal hyposecretory disorders such as cystic fibrosis–related meconium ileus and distal intestinal obstruction syndrome.

## Graphical abstract

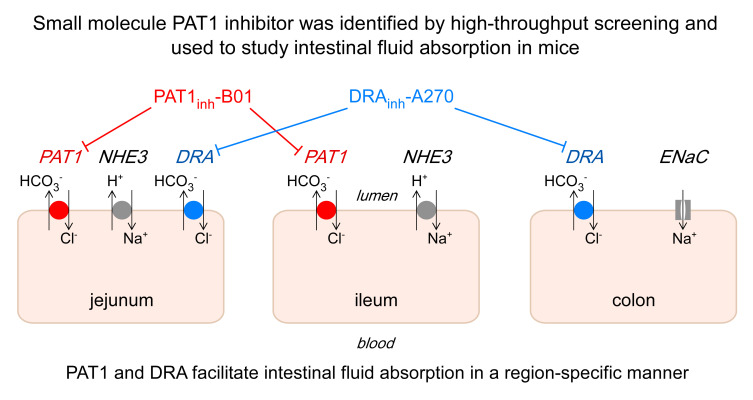

## Introduction

Electroneutral NaCl absorption in the small intestine, which drives fluid absorption, is mediated by the parallel actions of Na^+^/H^+^ and Cl^–^/HCO_3_^–^ exchangers. NHE3 is the dominant Na^+^/H^+^ exchanger in small intestine (1), and the NHE3 inhibitor tenapanor was recently approved for treatment of irritable bowel syndrome with constipation (2). SLC26A3 (also known as downregulated in adenoma [DRA]) and SLC26A6 (also known as putative anion transporter 1 [PAT1]) are the principal anion exchangers at the luminal plasma membrane of intestinal epithelial cells (3). Due to the lack of selective inhibitors, current understanding of the functional roles of PAT1 and DRA in intestinal fluid transport is mainly from studies in knockout mice. Some earlier studies suggested PAT1 as the predominant anion exchanger in small intestine (4, 5), while another study reported DRA expression in small intestine and loss of small intestinal Cl^–^ absorption in DRA-knockout mice (6). This difference may be the consequence of a combination of factors, including the lack of selective inhibitors, confounding effects of compensatory mechanisms in knockout mice, and possible species/strain differences.

A recent small-molecule screen, with follow-up compound optimization by medicinal chemistry, identified selective DRA inhibitors with nanomolar potency that blocked intestinal fluid absorption in mouse colon and were effective in experimental mouse models of constipation (7, 8). Here, to clarify the functional role of PAT1 in intestine, we identified and characterized a small-molecule PAT1-selective inhibitor, investigating its effects on intestinal fluid absorption in intestinal closed loops and testing its efficacy in a murine model of constipation, including additive action with a DRA inhibitor.

## Results

### PAT1 inhibitors identified by high-throughput screening.

**Figure 1:**
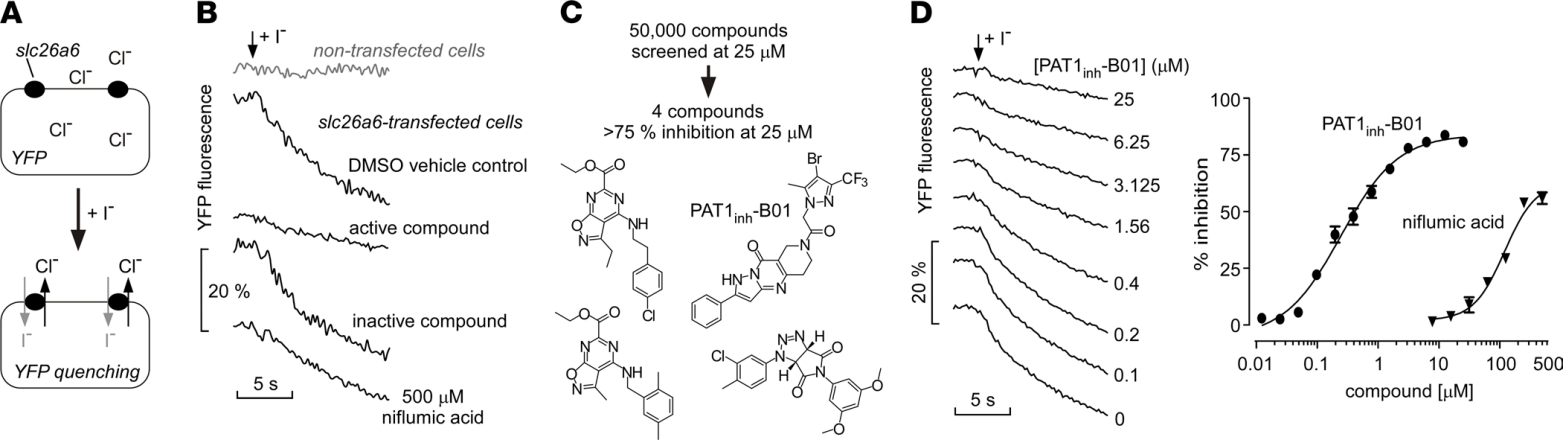
Small-molecule screen identifies PAT1 inhibitors. (**A**) Screening assay in which FRT cells coexpressing slc26a6 (PAT1) and a halide-sensing yellow fluorescent protein (YFP) were subject to an inwardly directed iodide (I^–^) gradient. PAT1-mediated influx of I^–^ in exchange for Cl^–^ reduces cytoplasmic YFP fluorescence. (**B**) Representative primary screening data from single wells of 96-well plates. NaI-containing solution (100 μl) was injected onto cells bathed in 100 μl of a NaCl-containing solution. YFP fluorescence curves shown for nontransfected cells (top curve) and for PAT1-expressing cells (bottom 4 curves). Cells were preincubated with vehicle (DMSO) control, test compound (examples of active and inactive compounds shown), or niflumic acid (positive control). (**C**) Summary of screening results (top), with chemical structures of active compounds identified in the primary screen (bottom). (**D**) Representative YFP fluorescence curves (left) and concentration dependence (right) for inhibition of PAT1-mediated Cl^–^/I^–^ exchange by PAT1_inh_-B01 and niflumic acid (mean ± SEM, *n* = 4).

A small-molecule screen was developed to identify PAT1 inhibitors utilizing transfected FRT cells expressing PAT1 (murine slc26a6) together with a halide-sensing cytoplasmic YFP (Figure 1A). Adherent cells in a 96-well format were subjected to an inward gradient of I^–^ to drive exchange of I^–^ for Cl^–^ and consequent quenching of the YFP fluorescence signal. Little fluorescence quenching was seen in nontransfected FRT cells in response to extracellular addition of I^–^ (Figure 1B). In PAT1-expressing cells, there was a large and rapid decrease in fluorescence in the absence of test compound or with an inactive test compound. A reduced rate of fluorescence quenching was seen with an active compound or with a high concentration of the nonselective anion transporter inhibitor niflumic acid.

Screening of 50,000 chemically diverse drug-like small molecules produced 4 compounds that at 25 μM inhibited PAT1-mediated Cl^–^/I^–^ exchange by more than 75% (Figure 1C). The most potent compound, PAT1_inh_-B01, inhibited PAT1 in a concentration-dependent manner with IC_50_ of 350 nM (Figure 1D), substantially better than that of 200 μM for niflumic acid. PAT1-mediated anion exchange was electroneutral in these studies, as shown by absence of inhibitor effect on short-circuit current in the PAT1-expressing FRT cells (data not shown) under conditions used previously (9) that produced large signals with anion channels CFTR and TMEM16A.

In addition to testing the 50,000 synthetic small molecules with diverse chemical structures, 699 additional compounds and their chemical analogs were screened that were found previously (7, 10, 11) to inhibit the related SLC26A anion exchangers DRA or pendrin. None of the DRA or pendrin inhibitor analogs inhibited PAT1-mediated Cl^–^/I^–^ exchange by more than 20% at 25 μM. Limited structure-activity relationship studies were done on PAT1_inh_-B01 using commercially available pyrazolo-pyrido-pyrimidin​one analogs. Of 269 analogs tested, 5 compounds showed PAT1 inhibition, albeit they were much weaker compared with PAT1_inh_-B01. Further characterization and in vivo studies were done with PAT1_inh_-B01 because of its better inhibition potency, compared with the other active compounds in Figure 1C, and its favorable solubility and drug-like chemical structure.

### Characterization of PAT1_inh_-B01 inhibition of PAT1 anion exchange.

**Figure 2:**
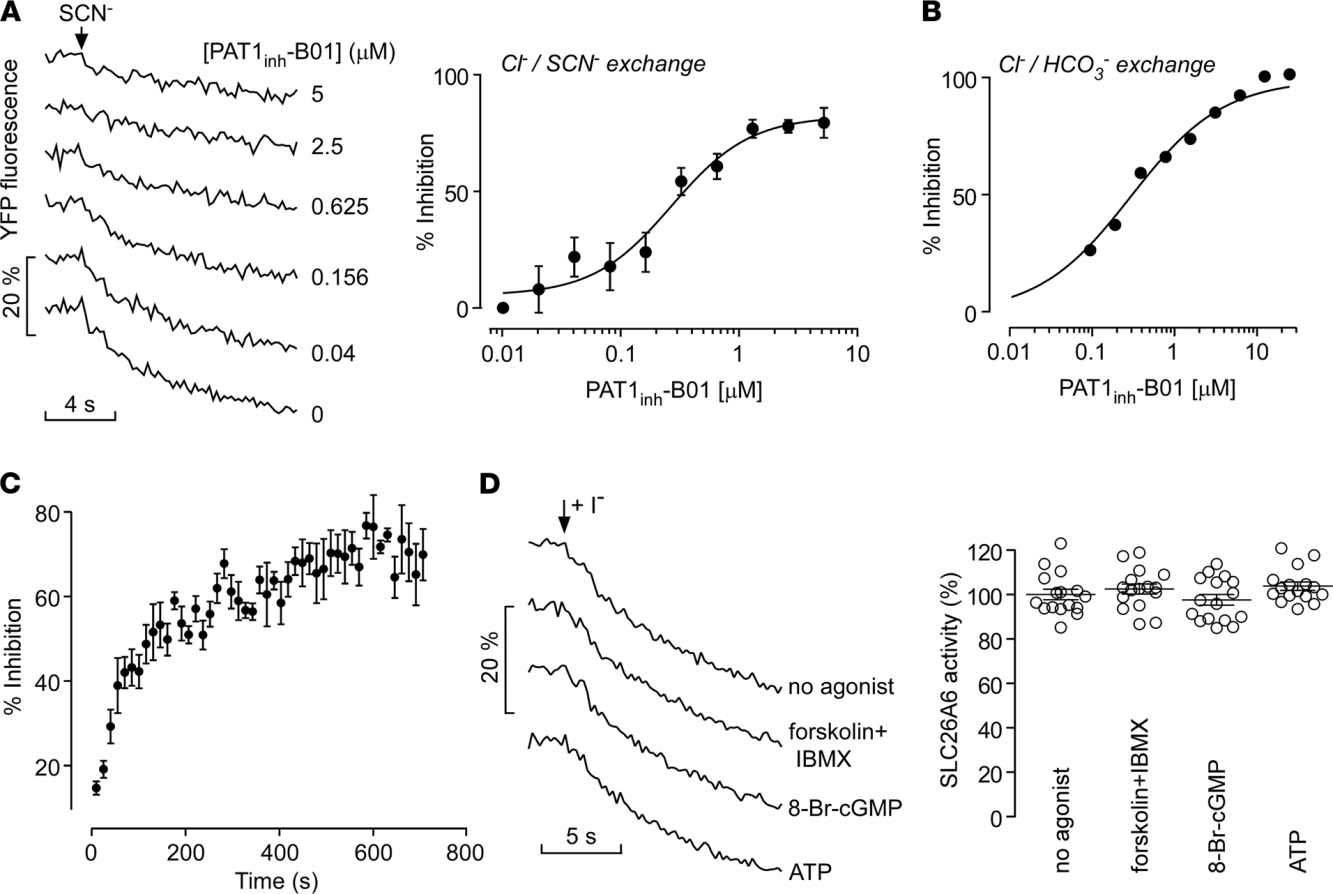
PAT1_inh_-B01 inhibition of PAT1-mediated anion exchange. (**A**) YFP fluorescence quenching curves (left), as in Figure 1B, for inhibition of PAT1-mediated exchange of Cl^–^ for SCN^–^, with concentration dependence of PAT1_inh_-B01 effect (right) (mean ± SEM, *n* = 3). (**B**) Inhibition of Cl^–^/HCO_3_^–^ exchange measured in BCECF-loaded FRT cells by PAT1_inh_-B01 (mean ± SEM, *n* = 16–32). (**C**) Time course of PAT1 inhibition following addition of 1 μM PAT1_inh_-B01 to cells (mean ± SEM, *n* = 3). (**D**) Effects of second messenger signaling on PAT1-mediated Cl^–^/I^–^ exchange. YFP fluorescence quenching curves (left) and summary data (right) following cell exposure to agonists of cAMP (20 μM forskolin + 200 μM IBMX), cGMP (100 μM 8-Br-cGMP) and Ca^+2^ (100 μM ATP), or vehicle control (1% DMSO). Mean ± SEM, *n* = 16 experiments per group, differences not significant by 1-way ANOVA and post hoc Newman-Keuls multiple comparisons test.

As PAT1 functions as a general monovalent anion exchanger, PAT1_inh_-B01 inhibition was studied for different modes of anion exchange. PAT1_inh_-B01 inhibited PAT1-mediated Cl^–^/SCN^–^ exchange with IC_50_ of 260 nM (Figure 2A). To measure Cl^–^/HCO_3_^–^ exchange, the time course of cytoplasmic pH was measured, using BCECF as a pH indicator, in response to a gluconate gradient to drive exchange of cytoplasmic Cl^–^ with extracellular HCO_3_^–^ producing cytoplasmic alkalinization (Figure 2B) as done before for DRA and pendrin (7, 10, 11). PAT1_inh_-B01 inhibited Cl^–^/HCO_3_^–^ exchange with IC_50_ of 290 nM. Figure 2C shows the time course of inhibition of PAT1-mediated Cl^–^/I^–^ exchange following addition of 1 μM PAT1_inh_-B01. PAT1 inhibition increased progressively with time, with 50% inhibition seen at approximately 3 minutes, suggesting an intracellular site of PAT1_inh_-B01 action. Inhibition was fully reversible following PAT1_inh_-B01 washout (data not shown).

An earlier study in intestinal Caco-2 and T84 cells suggested that PAT1 activity might be subject to regulation by secondary messengers such as cAMP (12). To test whether PAT1 is subject to direct regulation by cAMP, cGMP, or Ca^2+^, PAT1-mediated Cl^–^/I^–^ exchange was measured in the PAT1-expressing FRT cells following incubations with forskolin plus IBMX, 8-Br-cGMP, or ATP. These agonists did not significantly affect PAT1-mediated Cl^–^/I^–^ exchange (Figure 2D).

### PAT1_inh_-B01 selectivity.

**Figure 3:**
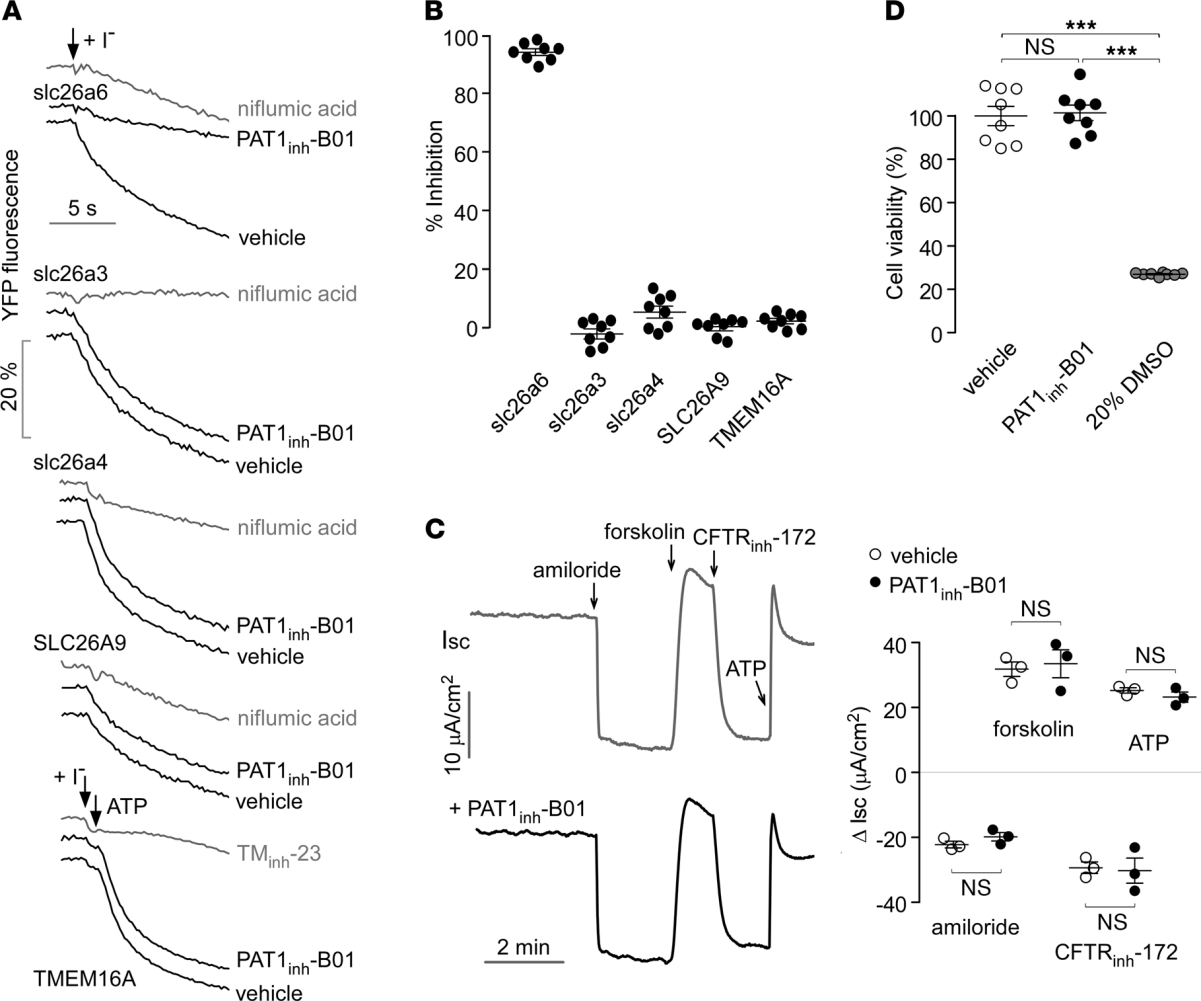
PAT1_inh_-B01 selectivity and cytotoxicity. (**A**) Selectivity of PAT1_inh_-B01 against related SLC26A family members and TMEM16A Cl^–^ channel. Cl^–^/I^–^ exchange or I^–^ influx measured in FRT cells coexpressing YFP and SLC26A isoforms or TMEM16A, with data shown for 25 μM PAT1_inh_-B01, DMSO vehicle control, and 500 μM niflumic acid (positive control for SLC26A transporters) or 10 μM TM_inh_-23 (positive control for TMEM16A). (**B**) Percentage inhibition of Cl^–^/I^–^ exchange or I^–^ influx from data in **A** (mean ± SEM, *n* = 8 experiments per condition). (**C**) (left) Short-circuit current (I_sc_) in well-differentiated HBE cells measured in the absence (top) and presence (bottom) of 25 μM PAT1_inh_-B01. Amiloride (20 μM), forskolin (20 μM), CFTR_inh_-172 (10 μM) and ATP (100 μM) were added as indicated. (right) Summary of I_sc_ changes in the absence and presence of PAT1_inh_-B01. Mean ± SEM, *n* = 3, differences not significant by Student’s *t* test. (**D**) Cell viability assayed by Alamar blue in FRT cells incubated for 48 hours with vehicle control (0.1% DMSO) or 10 μM PAT1_inh_-B01. 20% DMSO was used as positive control. Mean ± SEM, *n* = 8 wells per group, 1-way ANOVA and post hoc Newman-Keuls multiple comparisons test, ***P < 0.001.

PAT1_inh_-B01 selectivity was studied by testing its effects on related SLC26A family anion transporters and the Cl^–^ channel TMEM16A. These measurements used a similar Cl^–^/I^–^ exchange protocol, but FRT cells separately expressed each of the SLC26A family members or TMEM16A. As seen in original fluorescence quenching curves in Figure 3A and as summarized in Figure 3B, PAT1_inh_-B01 at a high concentration of 25 μM did not significantly inhibit slc26a3, slc26a4, SLC26A9, or TMEM16A. PAT1_inh_-B01 also did not affect the activities of slc26a1 and slc26a2 (Supplemental Figure 1; supplemental material available online with this article; https://doi.org/10.1172/jci.insight.147699DS1), other members of SLC26A family expressed in the intestine. PAT1_inh_-B01 selectivity was also tested by short-circuit current (I_sc_) in well-differentiated human bronchial epithelial (HBE) cells, which have a complex ion transport mechanism that includes cystic fibrosis transmembrane conductance regulator (CFTR), epithelial Na^+^ channel (ENaC), and Ca^+2^-activated chloride channels (CaCCs) (13). PAT1_inh_-B01 pretreatment had no significant effect on ENaC, CFTR, or CaCC activities, as evidenced by comparable I_sc_ responses to amiloride, forskolin/CFTR_inh_-172, and ATP, respectively (Figure 3C). Potential PAT1_inh_-B01 cytotoxicity was studied in FRT cells. Prolonged (48 h) PAT1_inh_-B01 incubation at a concentration of 10 μM did not cause cytotoxicity, as measured by the Alamar Blue assay (Figure 3D).

### PAT1_inh_-B01 blocks fluid absorption partially in mouse jejunum and completely in mouse ileum.

**Figure 4:**
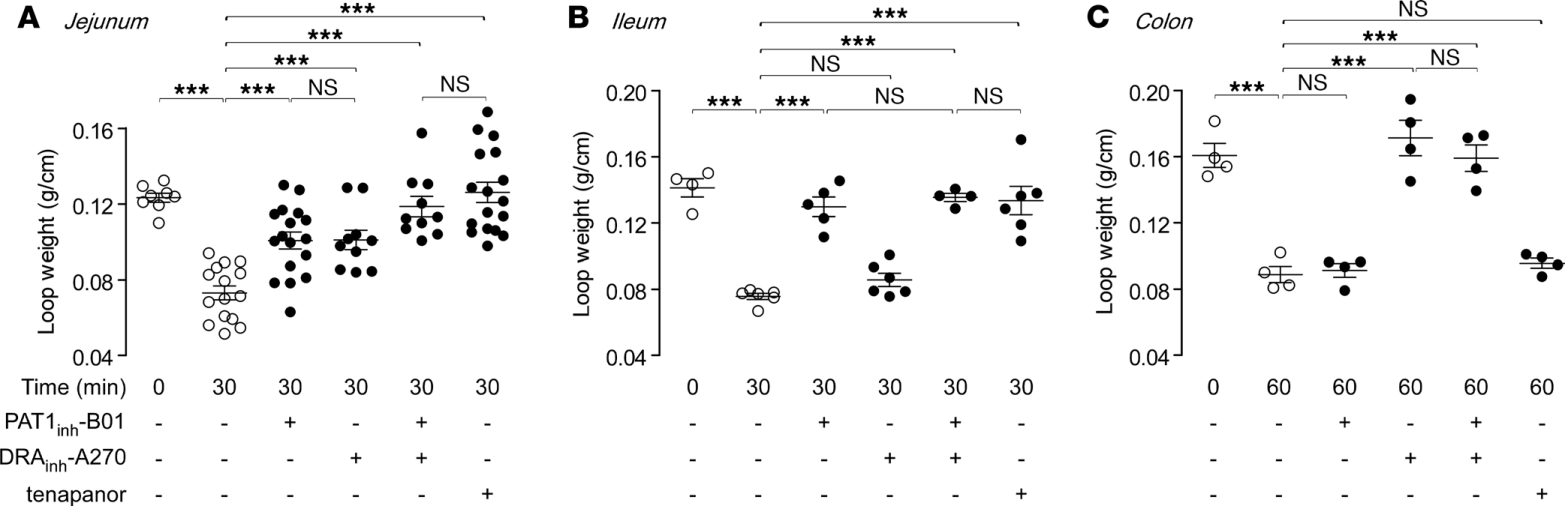
PAT1 inhibition blocks fluid absorption in mouse small intestine but not colon. (**A**) Effects of luminal PAT1_inh_-B01 (30 μM), DRA_inh_-A270 (10 μM), and tenapanor (10 μM), individually and together, on loop weight-to-length ratio at 30 minutes in mouse midjejunal closed loops (*n* = 8–17 loops per group). (**B**) Effects of the compounds in mouse ileal closed loops (*n* = 4–6 loops per group). (**C**) Effects of the compounds in mouse distal colonic closed loops (*n* = 4 loops per group). Mean ± SEM, 1-way ANOVA and post hoc Newman-Keuls multiple comparisons test, ***P < 0.001.

Closed intestinal loop studies in mice were done using the selective inhibitors PAT1_inh_-B01 and DRA_inh_-A270, individually and together, to investigate the contributions of PAT1 and DRA in intestinal fluid absorption. PAT1_inh_-B01 at 30 μM in the lumen of closed jejunal loops inhibited the decrease in loop weight-to-length ratio, a direct measure of fluid absorption, by approximately 50% (Figure 4A). In parallel studies, DRA_inh_-A270 at 10 μM also inhibited the reduction in loop weight-to-length ratio by approximately 50%. PAT1_inh_-B01 and DRA_inh_-A270, when administered together, produced >90% inhibition, comparable to that produced by the NHE3 inhibitor tenapanor at 10 μM. These results provide pharmacological evidence for comparable contributions of PAT1 and DRA to fluid absorption in mouse jejunum.

In ileal loops PAT1_inh_-B01 fully blocked fluid absorption, as seen from prevention of the reduction in loop weight-to-length ratio at 30 minutes, which was comparable to the tenapanor effect (Figure 4B). In contrast, DRA_inh_-A270 did not inhibit ileal fluid absorption, and PAT1_inh_-B01 and DRA_inh_-A270 together had comparable efficacy to that of PAT1_inh_-B01 alone. These results provide evidence that PAT1 is the main anion exchanger in the mouse ileum. To further characterize the effects of PAT1 inhibition, loop fluid pH was measured in ileal loops in the absence and presence of PAT1_inh_-B01. In control loops, luminal pH increased to greater than 8.0 at 30 minutes after injection of PBS (pH 7.4) (Supplemental Figure 2A), consistent with Cl^–^/HCO_3_^–^ exchange. The pH increase was prevented in loops containing PAT1_inh_-B01. These results suggest that the in vivo effect of PAT1_inh_-B01 on ileal fluid absorption is due to inhibition of PAT1-mediated Cl^–^/HCO_3_^–^ exchange. Although they probably do not have a significant contribution in fluid transport, sodium-dependent phosphate cotransporters, including NaPi-IIb, are highly expressed in mouse ileum (14). To investigate the potential contribution of phosphate transporters on the antiabsorptive effect of PAT1_inh_-B01 seen here, closed-loop experiments were done in ileum in the absence of luminal phosphate using HEPES-buffered saline. In this setting, PAT1_inh_-B01 effectively blocked fluid absorption (Supplemental Figure 2B).

In closed distal colonic loops PAT1_inh_-B01 did not inhibit the reduction in loop weight-to-length ratio, whereas, as reported previously (7), DRA_inh_-A270 completely prevented fluid absorption, as was also seen with PAT1_inh_-B01 and DRA_inh_-A270 together (Figure 4C). As reported previously (7), the NHE3 inhibitor tenapanor did not inhibit fluid absorption from mouse colonic loops.

**Figure 5:**
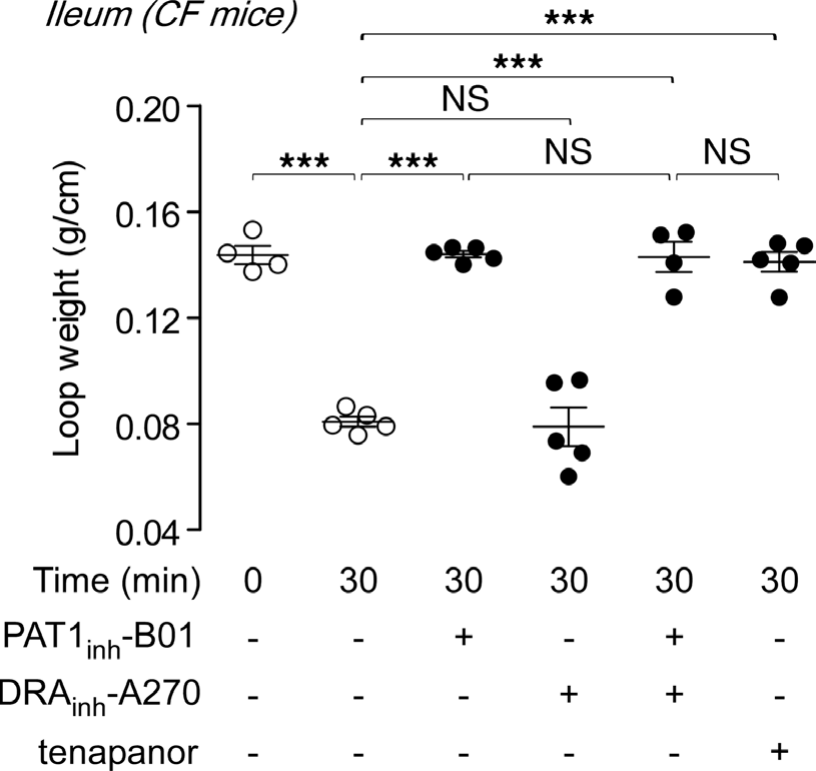
PAT1 inhibition blocks fluid absorption in ileum of cystic fibrosis mice. Effects of PAT1_inh_-B01 (30 μM), DRA_inh_-A270 (10 μM), and tenapanor (10 μM), as studied in Figure 4, in ileal closed loops in cystic fibrosis (CF) mice (homozygous F508del, *n* = 4–6 loops per group). Mean ± SEM, 1-way ANOVA and post hoc Newman-Keuls multiple comparisons test, ***P < 0.001.

Cystic fibrosis (CF) is associated with intestinal hyposecretory disorders, such as meconium ileus and distal intestinal obstructive syndrome (DIOS), which primarily affect the distal small intestine (ileum) (15). To test the potential utility of PAT1 inhibition in these conditions, closed-loop studies were done in ilea of CF mice (F508del homozygous). As found in wild-type mice, PAT1_inh_-B01 and tenapanor completely blocked fluid absorption from ilea of CF mice, whereas DRA_inh_-A270 had no effect (Figure 5). These results support the potential utility of PAT1 inhibitors for CF-related small intestinal disorders.

### Efficacy of PAT1_inh_-B01 and DRA_inh_-A270 in a loperamide model of constipation.

**Figure 6:**
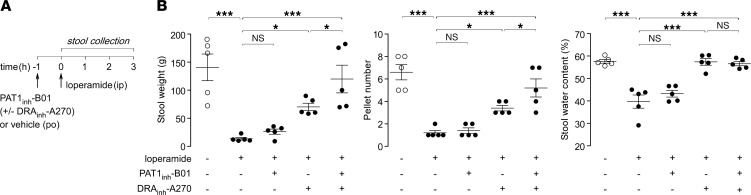
PAT1 and DRA inhibitors act in synergy in a loperamide model of constipation in mice. (**A**) Experimental protocol. (**B**) Effects of orally administered PAT1_inh_-B01 (10 mg/kg) and DRA_inh_-A270 (10 mg/kg), individually and together, on 3-hour stool weight (left), number of pellets (middle), and stool water content (right) in wild-type mice. Mean ± SEM, *n* = 5 mice per group, 1-way ANOVA and post hoc Newman-Keuls multiple comparisons test, *P < 0.05, ***P < 0.001.

The efficacy of PAT1_inh_-B01 was tested in loperamide-induced constipation model in mice (Figure 6A). Loperamide produces marked constipation in mice, as indicated by reduced weight, number of pellets, and water content for stool collected over 3 hours (Figure 6B). PAT1_inh_-B01 (10 mg/kg, oral) did not affect the stool parameters, whereas DRA_inh_-A270 (10 mg/kg, oral) normalized stool water content and increased stool weight and number of pellets, as reported previously (8). Coadministration of PAT1_inh_-B01 and DRA_inh_-A270 increased stool output significantly more than DRA_inh_-A270 alone. PAT1 inhibition alone thus did not affect stool output in mice, whereas it potentiated the laxative action of DRA inhibition.

## Discussion

A selective inhibitor of PAT1-mediated anion exchange was identified and characterized in order to investigate the relative contributions of PAT1 and DRA to fluid absorption in different regions of mouse intestine. Current understanding of the contributions of PAT1 and DRA in intestinal fluid absorption comes mainly from studies in knockout mice, as selective inhibitors have not been available. An early study showed strong PAT1 mRNA expression throughout the small intestine (duodenum, jejunum, and ileum) in mice, with no expression in the colon (5), whereas DRA mRNA was highly expressed in the colon with low-level expression throughout the small intestine. In mouse duodenum, Simpson et al. (4) reported comparable PAT1 and DRA mRNA expression, with Cl^–^/HCO_3_^–^ exchange activity reduced by 65%–80% and 30%–40% in PAT1- and DRA-knockout mice, respectively. Here, we did not investigate duodenal fluid absorption because the mouse duodenum is a short and fixed segment, precluding the generation of closed loops.

In jejunum, Walker et al. (6) reported that net Cl^–^ absorption was reduced by more than 80% in DRA-knockout mice but by less than 20% in PAT1-knockout mice. They concluded that DRA is the major apical anion exchanger in mouse jejunum, though found only modest DRA immunofluorescence. Another study reported approximately 50% reduction in net Cl^–^ absorption in jejunum in PAT1-knockout mice (16). The same group, using an in vivo perfusion technique, showed comparably reduced fluid absorption by approximately 30% in jejunum of both DRA- and PAT1-knockout mice (17). The functional data here using selective inhibitors are consistent with the latter studies and suggest comparable and complementary roles for PAT1 and DRA in jejunal fluid absorption in mice. We previously reported that the original, relatively low-affinity DRA inhibitor, DRA_inh_-A250, did not significantly affect fluid absorption in closed midjejunal loops in mice at 10 μM (7). In the current study, the 5-fold more potent DRA inhibitor DRA_inh_-A270 partially blocked fluid absorption in mouse jejunum at 10 μM, suggesting that DRA has a role in fluid absorption in this segment of the mouse intestine.

Whittamore et al. (18) investigated the relative contributions of PAT1 and DRA on ileal Cl^–^ absorption using knockout mice in a C57BL/6 genetic background. They reported that PAT1 knockout had no effect on net absorptive Cl^–^ flux across mouse ileum and that DRA knockout reduced absorptive Cl^–^ flux by 40%. The current study, using selective pharmacological inhibitors in CD1 mice, found that PAT1 is the main luminal anion exchanger responsible for fluid absorption in mouse ileum, with little contribution of DRA. This difference may be due to compensatory changes in knockout mice and/or strain differences (C57BL/6 vs. CD1). Further studies using selective PAT1 and DRA inhibitors in different animals and mouse strains may be informative. DRA is believed to be the major anion exchanger in mouse colon (3), as PAT1 mRNA is not expressed there (5). Consistent with these studies, PAT1 inhibition did not affect fluid absorption in mouse colonic loops under conditions in which DRA_inh_-A270 fully inhibited fluid absorption.

Loss of prosecretory CFTR activity is thought to be the principal cause of gastrointestinal disorders in CF, including chronic constipation, which has up to 47% prevalence, as well as the less common but more serious meconium ileus and DIOS (15, 19). We previously showed efficacy of DRA inhibition in a loperamide constipation model in wild-type and CF mice (7). Here, we found additive antiabsorptive effects of PAT1_inh_-B01 and DRA_inh_-A270 in mouse jejunum, suggesting potential synergy of PAT1 and DRA inhibition for treatment of constipation. This is also supported by the increased stool output when PAT1 and DRA inhibitors were used together in a constipation model in mice. Meconium ileus and DIOS are caused by excessive dehydration of intestinal luminal contents primarily in ileum (15), where PAT1 has the predominant role in fluid absorption, as found here. There is limited information in the literature on PAT1 expression in CF mouse intestine. Early studies in CF mice focusing on duodenum reported varying results for PAT1 expression. Knauf et al. (20) showed that PAT1 expression is reduced by 40% in duodenum of CF mice compared with that in wild-type mice, whereas Simpson et al. (21) showed similar PAT1 mRNA expression in the duodena of CF and wild-type mice. Our closed loop studies suggest comparable PAT1 activity in wild-type and CF mouse ilea, based on similar reductions in loop weight-to-length ratio and similar efficacy of PAT1_inh_-B01. The results here suggest the potential efficacy of PAT1 inhibitors for gastrointestinal conditions that affect small intestine. PAT1 inhibitors, by blocking fluid absorption in distal small intestine, are predicted to hydrate ileal luminal contents, which might prevent or treat obstruction in meconium ileus and DIOS.

We note that PAT1 is expressed in several organs outside of the intestine, including kidneys, heart, and pancreatic ducts, raising the issue of potential adverse effects such as arrhythmias (22); however, problems related to extraintestinal PAT1 expression have not been reported so far in humans with *SLC26A6* mutations. Development of a nonabsorbable PAT1 inhibitor (similar to the NHE3 inhibitor tenapanor; ref. 2), may be useful in this regard. A concern with chronic PAT1 inhibition is the hyperoxaluria and calcium oxalate nephrolithiasis seen in PAT1-knockout mice due to impaired intestinal oxalate secretion (23); however, *SLC26A6* variants in humans are not associated with hyperoxaluria or nephrolithiasis (24). Thus, it is unclear whether hyperoxaluria would be a side effect of chronic PAT1 inhibitor treatment in humans. These potential side effects would be of lesser concern for the short-term pharmacological PAT1 inhibition, as might be used for meconium ileus and DIOS.

Although classified as a solute carrier family, there is limited sequence similarity between various slc26a isoforms (25). In intestinal epithelial cells, 4 slc26a isoforms (slc26a1, slc26a2, slc26a3, and slc26a6) are mainly expressed, with apparently different expression levels in different segments (26). Here, we provide functional evidence that PAT1_inh_-B01 does not affect ion transport mediated by other major intestinal slc26a isoforms, ion channels, or transporters. The observed in vivo effect of PAT1_inh_-B01 is therefore due to its selective action on PAT1.

With regard to the compound PAT1_inh_-B01, there are no previous biological reports on the pyrazolo-pyrido-pyrimidinone scaffold, though a class of pyrimido-​pyrazolo-quinolines that shares some structurally similarity was reported to restore chemosensitivity of colonic cancer cells in vitro (27). Pyrazolo-pyrido-pyrimidinone scaffolds can be prepared in 3–6 steps from commercially available starting chemicals. PAT1_inh_-B01 has drug-like physiochemical properties, including the presence of multiple hydrogen bond acceptors, with partition coefficient (aLogP), molecular weight, and topological polar surface areas of 3.3, 535, and 88.3 Å^2^, respectively. Further medicinal chemistry efforts may produce analogs with greater potency.

In conclusion, to our knowledge the PAT1-selective inhibitor identified herein is a novel research tool that was used to resolve the relative functional contributions of PAT1 and DRA on fluid absorption in different intestinal segments. PAT1 inhibitors should be useful for further physiological studies of intestinal Cl^–^ and HCO_3_^–^ transport as well as PAT1 function in other organs where it is expressed. PAT1 inhibitors are also potential drug candidates for certain intestinal disorders, including CF-related meconium ileus and DIOS.

## Methods

### Chemicals.

All chemicals were purchased from MilliporeSigma unless otherwise specified. DRA_inh_-A270 was synthesized and purified as described previously (8).

### cDNA constructs.

A vector containing (murine) slc26a6 cDNA was purchased from Origene (cDNA clone MC202861) and confirmed by sequence analysis. The slc26a6 cDNA was excised by digestion with *Spe*I and *Not*I and subcloned into the pLVX-IRES-mCherry lentiviral expression cassette (Clontech) at *Nhe*I and *Not*I sites. To generate a cell line for screening, slc26a6 lentiviral particles were produced in HEK293 cells using the pRSV-Rev, pMDLg/pRRE, and pMD2.G packaging vectors (Addgene, deposited by Didier Trono, plasmids 12253, 12251, and 12259) and used to transduce Fischer rat thyroid (FRT) cells previously transduced with lentivirus expressing EYFP-H148Q/I152L/F46L (YFP) (7). FRT cells were obtained from the University of California, San Francisco, Cystic Fibrosis Drug Discovery Core Center. For lentivirus production, HEK293T cells (ATCC, CRL-3216) were transfected with pLVX-IRES-mCherry-slc26a6, pRSV-Rev, pMDLg/pRRE, and pMD2.G using NanoFect transfection reagent (Alstem) per the manufacturer’s instructions. After 1 day, the medium serum content was reduced to 2.5%, and after 2 days, the medium was harvested, centrifuged (5 min, 5000*g*) and concentrated using Microcon centrifugal filters (MilliporeSigma) with 10-kDa molecular-weight cutoff.

### Cell culture.

Clonal FRT cell lines were generated by limited dilution, examined by fluorescence microscopy to confirm mCherry and YFP expression, and functionally characterized to confirm slc26a6 activity to generate the cell line FRT-YFP-slc26a6. FRT cell lines coexpressing YFP and slc26a3, slc26a4, SLC26A9, or TMEM16A were previously described (7, 11, 28). FRT cells were cultured with Kaign’s modified Ham’s F12 medium supplemented containing 10% FBS, 2 mM L-glutamine, 100 U/ml penicillin, 100 μg/ml streptomycin, 18 μg/ml myo-inositol, and 45 μg/ml ascorbic acid, with appropriate selection antibiotics. Well-differentiated HBE cells were cultured at an air-liquid interface on inserts as described previously (13). HBE cells were used for short-circuit current experiments 3 weeks after plating when they formed a tight epithelium (*R*_TE_ > 1000 Ω cm^2^).

### High-throughput screening.

A collection of approximately 50,000 diverse, drug-like synthetic small molecules (ChemDiv) was screened at a concentration of 25 μM using a Beckman Coulter BioMek FX liquid handling platform with FLUOstar OMEGA plate readers (BMG Labtech). FRT-YFP-slc26a6 cells were plated in 96-well black-walled, clear-bottom plates (Corning Life Sciences) at a density of 20,000 cells/well and cultured for 48 hours until confluent. Cells were washed twice in PBS and incubated for 10 minutes prior to assay in 100 μl PBS containing test compounds. For assay of slc26a6 function, baseline cellular fluorescence was measured for 2 seconds, after which 100 μl NaI-substituted PBS (140 mM NaCl replaced by 140 mM NaI) was added by syringe pump to drive Cl^–^/I^–^ exchange. The initial rate of Cl^–^/I^–^ exchange was determined by single exponential regression of the fluorescence time course. All assay plates contained wells with negative (1% DMSO) and positive (500 μM niflumic acid) controls. For structure-activity relationship studies, analogs of active compounds were purchased from ChemDiv.

### Selectivity studies and additional assessment of slc26a6 activity.

YFP-based cellular assays of slc26a3, slc26a4, SLC26A9, and TMEM16A function were performed as described previously (7). Slc26a1 and slc26a2 assays were done in transfected HEK 293 cells (see Supplemental Methods for details). Niflumic acid was used as a positive control for SLC26A isoforms (7). For TMEM16A, the selective small-molecule inhibitor TM_inh_-23 was used as a positive control (28). The activities of ENaC, CFTR, and CaCC were determined using well-differentiated HBE cells as described previously (13). Cl^–^/HCO_3_^–^ exchange was measured as described previously (7, 10, 11). Briefly, cell cytoplasm was labeled with the pH-sensitive fluorescent dye BCECF-AM (Invitrogen), and Cl^–^/HCO_3_^–^ exchange was measured in response to addition of a gluconate-containing solution to drive exchange of cytoplasmic Cl^–^ for extracellular HCO_3_^–^, producing cytoplasmic alkalinization.

### Cellular toxicity.

FRT cells were plated in black-walled, clear-bottom tissue culture plates at a density of 20,000 cells/well. After 24 hours in culture, cells were incubated with 10 μM PAT1_inh_-B01, 0.1% DMSO (vehicle control), or 20 % DMSO (positive control) for 48 hours for assay of cell viability using Alamar Blue (Thermo Fischer Scientific) according to the manufacturer’s instructions.

### Animals.

Wild-type CD1 mice (male and female, age 10–16 weeks) and CF mice (homozygous CFTR F508del, male and female, age 12–20 weeks) were bred in the University of California, San Francisco, Laboratory Animal Resource Center. Animals were housed in communal cages with standard rodent chow and water available ad libitum.

### Closed-loop studies of intestinal fluid absorption.

For fluid absorption studies in small intestine, mice were given free access to 5% dextrose in water but not solid food for 24 hours before experiments. Closed midjejunal and ileal loops were prepared and isolated as described previously (29). Mice were anesthetized with isoflurane, and body temperature was maintained during surgery at 36°C–38°C using a heating pad. A small abdominal incision was made to expose small intestine for isolation of 3–4 closed small intestinal loops (jejunum or ileum, length 2–3 cm) with sutures. Loops were injected with 100 μL PBS (pH 7.4, 137 mM NaCl, 2.7 mM KCl, 8 mM Na_2_HPO_4_, 1.8 mM KH_2_PO_4_, 1 mM CaCl_2_, 0.5 mM MgCl_2_) containing 30 μM PAT1_inh_-B01, 10 μM DRA_inh_-A270, or 10 μM tenapanor (MedChemExpress) (or vehicle, 0.1% DMSO in PBS). In some experiments, 30 μM PAT1_inh_-B01 and 10 μM DRA_inh_-A270 were used together. In some experiments to rule out effects of sodium-dependent phosphate transport, the loop lumen contained HEPES-buffered saline (pH 7.4, 137 mM NaCl, 4.5 mM KCl, 10 mM HEPES, 1 mM CaCl_2_, 0.5 mM MgCl_2_). The abdominal incision was closed with sutures, and mice were allowed to recover from anesthesia. Intestinal loops were removed at 0 and 30 minutes (in separate mice), and loop length and weight were measured to quantify fluid absorption. In parallel experiments, CF mice were used and loop studies were done as described above. In some experiments, loop fluid was emptied with a syringe, and pH was measured immediately using AB15 pH meter after brief centrifugation at 2000 *g* for 5 minutes at 4°C.

For fluid absorption studies in colon, mice were given free access to 5% dextrose in water but not solid food for 48 hours. Mice were treated with 500 μL mineral oil twice a day rectally (final dose 16 h before surgery) during this period to evacuate the colon. Closed distal colonic loops (length 1.5–2 cm, 1 loop per mouse) were isolated as described previously (7). Loops were injected with 100 μL PBS with 30 μM PAT1_inh_-B01, 10 μM DRA_inh_-A270, or 10 μM tenapanor (or vehicle). In some experiments, 30 μM PAT1_inh_-B01 and 10 μM DRA_inh_-A270 were used together. Colonic loops were removed at 0 and 60 minutes (in separate mice), and loop length and weight were measured to quantify fluid absorption.

### Loperamide model of constipation.

Wild-type CD1 mice were administered loperamide (0.3 mg/kg, intraperitoneally) in PBS containing 5% ethanol (0.1 mg/mL final concentration) to induce constipation (9, 30). PAT1_inh_-B01 and DRA_inh_-A270, individually or together (10 mg/kg each, in saline containing 5% DMSO and 10% Kolliphor HS 15), or vehicle control were administered by oral gavage 1 hour before loperamide. After loperamide injection mice were placed individually in metabolic cages with free access to food and water. Stool samples were collected for 3 hours, and total stool weight and number of fecal pellets were quantified. To measure stool water content, stool samples were dried at 80°C for 24 hours and water content was calculated as follows: (wet weight – dry weight)/wet weight (9, 30).

### Statistics.

Experiments with 2 groups were analyzed using 2-tailed Student’s *t* test; for 3 or more groups, analysis was done with 1-way ANOVA and post hoc Newman-Keuls multiple comparisons test. For all analyses, *P* values of less than 0.05 were considered statistically significant.

### Study approval.

Protocols were approved by the University of California, San Francisco, Institutional Animal Care and Use Committee.

## Author contributions

OC and ASV conceived the study. OC, PMH, JATT, and AAR performed the experiments. OC, PMH, and JATT analyzed the data. OC, PMH, and ASV wrote the paper.

## Supplementary Material

Supplemental data

## Version 1 06/08/2021

Electronic publication
