# Supplementary material for: SLC26A6-selective inhibitor identified in a small-molecule screen blocks fluid absorption in small intestine
Source: JCI Insight. 2021 Jun 8;6(11):e147699. doi: 10.1172/jci.insight.147699 (PMC8262356; doi:10.1172/jci.insight.147699)
Supplement: Supplemental data [file jciinsight-6-147699-s007.pdf]

**Supplementary Material**

***Slc26a1 and slc26a2 cDNA constructs***

The complementary DNA sequences of slc26a1 and slc26a2 were generated using gBLOCK gene fragments (Integrated DNA technology, Coralville, IA) in the plasmid vector pIRESpuro3 (Takara, San Jose, CA). In brief, the 5' region of slc26a1 (which consists of a ~1150 bp gBlock with a blunt 5' end and a NheI site at the 3' end) was cloned into pIRESpuro3 at EcoRV / NheI sites. Subsequently, the 3' end of slc26a1 (which consists of a ~990 bp gBlock with NheI site at the 5' end, a silent mutation at valine 446 to remove a second NheI site, and a NotI site at the 3' end) was added to the 5' end of slc26a1 at NheI / NotI sites. The 5' region of slc26a2 (which consists of a ~1600 bp gBlock with a 5' NheI site and an EcoRI site at the 3' end) was cloned into pIRESpuro3 at NheI / EcoRI sites. Subsequently, the 3' end of slc26a2 (which consists of a ~600 bp gBlock with the intrinsic EcoRI site at the 5' end, and a NotI site at the 3' end) was added to the 5' end of slc26a2 at EcoRI / NotI sites. Full-length slc26a2 cDNAs were subsequently transferred as NheI / NotI fragments to a modified pIRESpuro3 plasmid in which the puromycin resistance gene was replaced with the EYFP-H148Q/I152L/F46L coding sequence (pIRES-HIF, Haggie et al., JCI Insight 2018). To generate the pIRES-HIF version of slc26a1, a NotI / XbaI fragment in pIRES-puro3-slc26a1 (containing the internal ribosome entry site and puromycin resistance gene) was replaced with a NotI / XbaI fragment from pIRES-HIF. cDNA constructs were confirmed by sequence analysis.

***Slc26a1 and slc26a2 functional assays***

To measure slc26a1 and slc26a2 exchange activity, 293 T/17 cells were grown on poly-D-lysine coated 18-mm diameter glass coverslips and transiently transfected with pIRES-HIF vectors using NanoFect Transfection reagent (Alstem Cell Advancements, Richmond, CA) using manufacturer's guidelines. Two days after transfection cells were washed twice with PBS, transferred to a custom cell chamber, and bathed in 250 μl

PBS. Cells were imaged on a stage of a TE2000 microscope (Nikon, Melville, NY) equipped with a C9100 EM-CCD (Hamamatsu, San Jose, CA), XCite light source (Excelitas, Waltham, MA), Nikon 20× NA 0.75 S Fluor objective, Uniblitz shutter (Vincent Associates, Rochester, NY) and B-2E/C filter set (Chroma, Bellows Fall, VT). To assay slc26a1 and slc26a2 function, 200-ms duration cell images were acquired continually, and exchange activity was initiated with the addition of 750 μl sodium iodide-substituted PBS. Background-corrected, area-integrated single cell fluorescence was calculated for determination of slc26a1 / slc26a2 activity from the rate of fluorescence quenching. To test inhibitor selectivity cells were incubated with 25 μM $PAT1_{inh}$-B01 for 10 min prior to assays. Controls experiments using cells infected with EYFP-H148Q/I152L/F46L alone were used to determine intrinsic iodide leak in 293 T/17 cells.

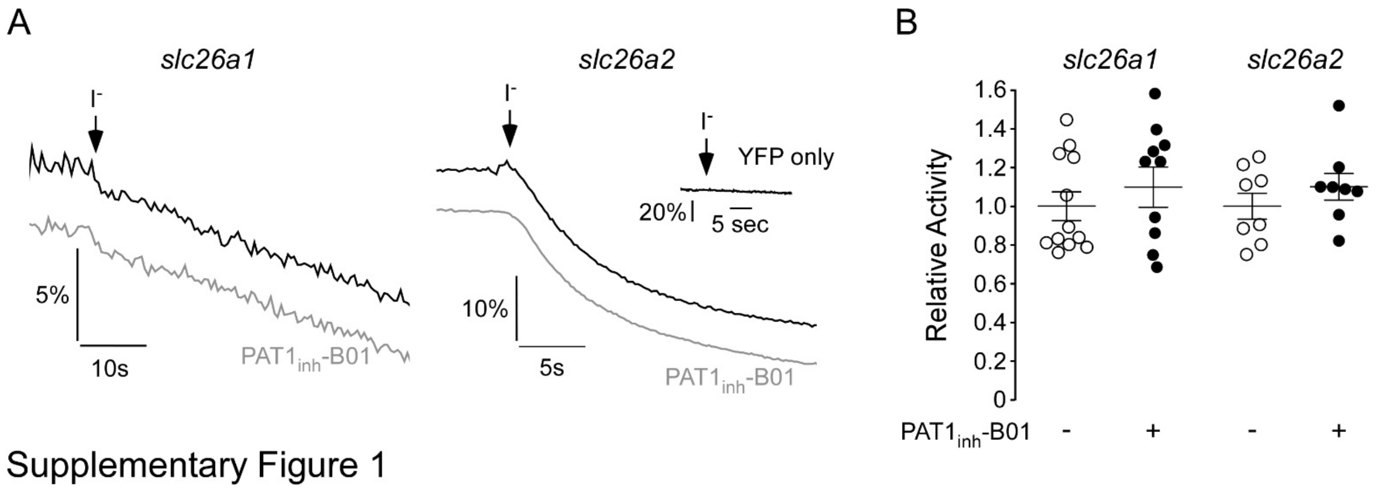


**Supplementary Figure 1. PAT1inh-B01 selectivity against slc26a1 and slc26a2. A.** 293 T/17 cells were transiently transfected with a plasmid to co-express slc26a1 or slc26a2 and halide-sensitive YFP. $Cl^-/I^-$ exchange activity showed without (*top*) and with (*bottom*) 25 μM PAT1inh-B01. Inset (right) shows fluorescence from 293 T/17 cells expressing YFP alone. **B.** Summary data for PAT1inh-B01 selectivity against slc26a1 and slc26a2. n=8-12 experiments per condition, mean ± S.E.M, differences not significant.

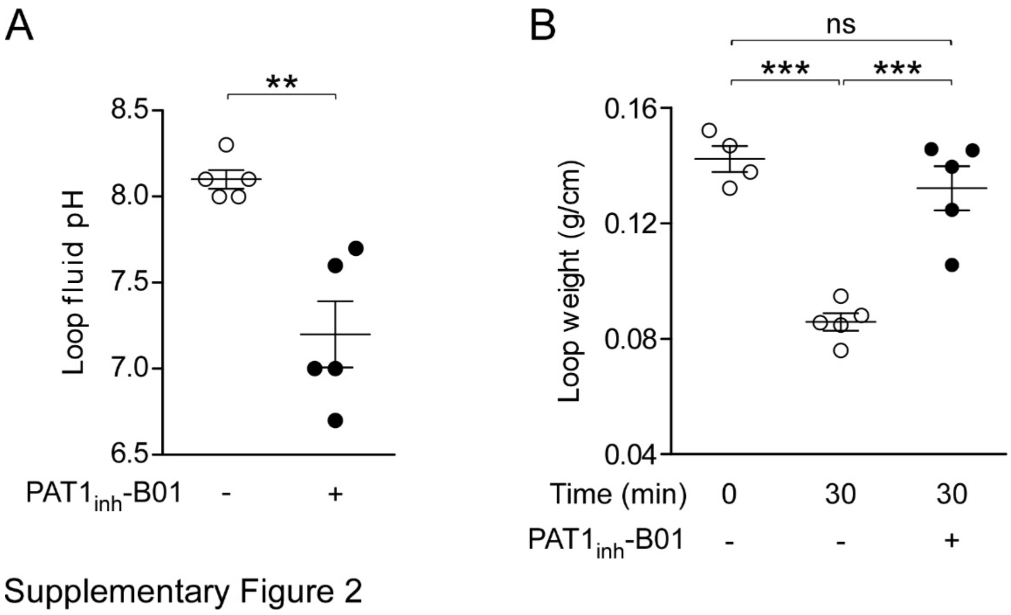


**Supplementary Figure 2. A.** Loop fluid pH in ileal closed loops at 30 min after injection of 100 μL phosphate-buffered saline (pH 7.4) without and with 30 μM $PAT1_{inh}$-B01. **B.** Loop weight/length ratio of ileal closed loops injected with 100 μl HEPES-buffered saline at 0 min without and with 30 μM $PAT1_{inh}$-B01. n=4-5 loops per group, mean ± S.E.M., **p<0.01, ***p<0.001, ns: not significant. Student's t-test for panel A, one-way analysis of variance and post hoc Newman-Keuls multiple comparisons test for panel B.
